# LC-QTOF-MS and ^1^H NMR Metabolomics Verifies Potential Use of Greater Omentum for *Klebsiella pneumoniae* Biofilm Eradication in Rats

**DOI:** 10.3390/pathogens9050399

**Published:** 2020-05-21

**Authors:** Joanna Teul, Stanisław Deja, Katarzyna Celińska-Janowicz, Adam Ząbek, Piotr Młynarz, Piotr Barć, Adam Junka, Danuta Smutnicka, Marzenna Bartoszewicz, Jerzy Pałka, Wojciech Miltyk

**Affiliations:** 1 Department of Pharmaceutical and Biopharmaceutical Analysis, Medical University of Bialystok, 15-089 Bialystok, Poland; katarzyna.siemionow@gmail.com (K.C.-J.); wojciech.miltyk@umb.edu.pl (W.M.); 2 Faculty of Chemistry, Opole University, 45-040 Opole, Poland; stanislaw.deja@pwr.edu.pl; 3 Department of Chemistry, Wroclaw University of Technology, 50-370 Wroclaw, Poland; adam.zabek@port.org.pl (A.Z.); piotr.mlynarz@pwr.edu.pl (P.M.); 4 Department of Vascular, General and Transplantation Surgery, Wroclaw Medical University, 50-367 Wroclaw, Poland; barc.wroclaw@gmail.com; 5 Department of Pharmaceutical Microbiology and Parasitology, Wroclaw Medical University, 50-367 Wroclaw, Poland; adam.junka@umed.wroc.pl (A.J.); danuta.ruranska-smutnicka@umed.wroc.pl (D.S.); marzenna.bartoszewicz@umed.wroc.pl (M.B.); 6 Department of Medicinal Chemistry, Medical University of Bialystok, 15-089 Bialystok, Poland; pal@umb.edu.pl

**Keywords:** metabolomics, *Klebsiella pneumoniae*, microbial biofilm eradication, surgery, implants

## Abstract

Bacterial wound infections are a common problem associated with surgical interventions. In particular, biofilm-forming bacteria are hard to eradicate, and alternative methods of treatment based on covering wounds with vascularized flaps of tissue are being developed. The greater omentum is a complex organ covering the intestines in the abdomen, which support wound recovery following surgical procedures and exhibit natural antimicrobial activity that could improve biofilm eradication. We investigated changes in rats’ metabolome following *Klebsiella pneumoniae* infections, as well as the greater omentum’s ability for *Klebsiella pneumoniae* biofilm eradication. Rats received either sterile implants or implants covered with *Klebsiella pneumoniae* biofilm (placed in the peritoneum or greater omentum). Metabolic profiles were monitored at days 0, 2, and 5 after surgery using combined proton nuclear magnetic resonance (^1^H NMR) and high performance liquid chromatography quadrupole time-of-flight tandem mass spectrometry (LC–QTOF-MS) measurements of urine samples followed by chemometric analysis. Obtained results indicated that grafting of the sterile implant to the greater omentum did not cause major disturbances in rats’ metabolism, whereas the sterile implant located in the peritoneum triggered metabolic perturbations related to tricarboxylic acid (TCA) cycle, as well as choline, tryptophan, and hippurate metabolism. Presence of implants colonized with *Klebsiella pneumoniae* biofilm resulted in similar levels of metabolic perturbations in both locations. Our findings confirmed that surgical procedures utilizing the greater omentum may have a practical use in wound healing and tissue regeneration in the future.

## 1. Introduction

*Klebsiella pneumoniae* belongs to the *Enterobacteriaceae* family and is a Gram-negative, non-motile, encapsulated bacteria found in the normal microbiome of the mouth, skin, and intestines [1]. In recent years, *Klebsiella pneumoniae* has become an important pathogen in nosocomial infections [2,3,4]. In the USA, the estimated number of such infections was 687,000 in 2015, with close to 72,000 associated deaths [5]. In Europe, 8.9 million patients suffered from nosocomial infections in 2016 [6], and it was estimated that every year close to 91,130 of deaths occur as a direct consequence of these infections [7]. Gram-negative *Enterobacteriaceae* are often a source of chronic bone inflammation derived after surgical treatment of severe injuries of motor organs. In particular, metal or silicone implants used for tissue or bone reconstruction serve as a perfect scaffold for microbial biofilm establishment [8]. Such biofilm infections are hard to eradicate due to their inherent tolerance to antibiotics and physical cleansing methods leading to chronic wound infections [9]. Therefore, effective alternative approaches for prevention and biofilm eradication related to chronic and device-associated infections are critically needed. An alternative strategy is to cover wounds with different types of pedicled or vascularized flaps [10,11]. According to clinical reports, the greater omentum is one of the most promising candidates for flaps with angiogenic and antibacterial potential [12].

The abdominal cavity is lined with the peritoneum, a complex serous organ with a variety of functions such as internal organ support through blood vessels, lymphatic vessels, and nerves. The area of the peritoneum is greater than the surface of the abdominal cavity, as the peritoneum consists of number of different folds and flaps. Among these, the greater omentum is special due to its ability to aid healing. The greater omentum is a mesothelial sheet-like tissue attached to the greater curvature of the stomach. It predominantly contains adipocytes embedded in a loose connective tissue as well as aggregates of mononuclear phagocytic cells. The greater omentum develops in the eighth week of gestation from the dorsal mesogastrium. This mobile structure possesses a remarkable power for aiding tissue repair due to excellent blood supply, as well as numerous lymphatic vessels. The healing potential of the omentum has been utilized clinically for many decades through transposing the omental pedicle or flap to injured organs (omental transposition) [13,14,15]. The greater omentum is known for its diverse functions such as controlling the spread of inflammation, as well as promoting revascularization, tissue reconstruction, and regeneration [16]. It has been used to treat infections such as mediastinitis, chronic cranial osteomyelitis, and deep sternal wound infection [17]. It has also been used for chest wall reconstruction [18,19]; to repair bronchopleural fistula; to close perforations in the gastrointestinal tract; to aid homeostasis during liver resections; in neurosurgery [20,21]; and for revascularization of ischemic brain, myocardium, and lower and upper extremities [22,23,24]. The greater omentum has also been used to support regeneration of neurons across a transected spinal cord in experiments in cats and also in one human patient [25,26,27], resulting in the unexpected recovery of limb function. The production of a variety of growth factors by the omentum provides a possibility to sustain transplanted pancreatic islets, expand cultured hepatocytes, and grow embryonic kidney and pancreas anlagen into adult organs [28,29,30,31].

The omentum possesses a natural ability for sensing injured sites in the abdominal cavity and firmly adhering to them. Similarly, when a foreign body is sensed, the omentum extends and expands to encapsulate the object as if to protect the adjoining internal organs from contact with it. Contact with a foreign body activates omental cells and they become a rich source of growth factors, expressing pluripotent stem cell markers [32]. Moreover, activated omentum cells after engrafting in injured tissues function as stem cells [32]. Numerous publications indicate beneficial effects of omentum transposition in case of healing acute injuries but they consist mainly of clinical examples. However, studies exploring the mechanisms, by which the omentum exerts curing effects, are still rare.

Modern metabolomics allows for simultaneous analysis of numerous compounds in biological samples that should reflect metabolic processes occurring in the organism. Changes in metabolite levels are rapid, and have a potential to be used as disease biomarkers or personalized patient recovery monitoring. In particular, urine is an easily accessible biofluid that reflects not only whole body but also microbial metabolism, and therefore can be potentially used for tracing bacterial activity. However, due to high variability in physicochemical properties of different metabolites, there is no single analytical approach covering all metabolites. In order to increase metabolite coverage, utilization of complementary analytical techniques, such as nuclear magnetic resonance (NMR) and high-performance liquid chromatography combined with mass spectrometry (LC–MS), is recommended [33].

In this study, we investigated metabolic perturbations that occur upon implant grafting in the greater omentum or peritoneum. We implanted sterile and *Klebsiella pneumoniae* biofilm-covered implants in rats and followed changes in their urine metabolite composition over time using two complementary analytical techniques: high performance liquid chromatography quadrupole time-of-flight tandem mass spectrometry detector (LC–QTOF–MS) and proton nuclear magnetic resonance (^1^H NMR). Urine metabolic fingerprinting revealed metabolic changes associated not only with surgical intervention but also specific to colonized implants. On the basis of these results, we aimed to confirm the ability of the greater omentum to eradicate *Klebsiella pneumoniae* biofilm and its potential use in clinical procedures for wound healing.

## 2. Results and Discussion

**Figure 1:**
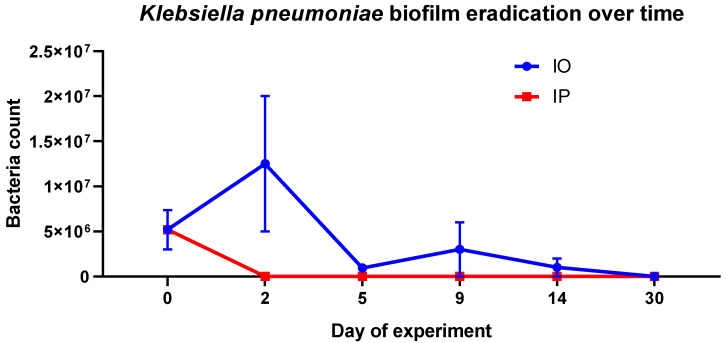
The number of bacteria left on the implant withdrawn from rats of IO (infected omentum) (blue dots) and IP (infected peritoneum) (red boxes) groups according to the day of experiment. Data are presented as mean and standard error.

Rats that underwent surgery received implants (sterile or colonized with *Klebsiella pneumoniae* biofilm) that were placed either in the peritoneum (SP—sterile peritoneum, IP—infected peritoneum) or in the greater omentum (SO—sterile omentum, IO—infected omentum). Control rats did not undergo surgical procedures in order to control for the effect of surgery on urine metabolome. Prior to metabolomics analysis we evaluated whether treatments were successful in biofilm eradication by quantifying the number of bacteria left on the implants. Although our study was focused on the initial metabolic changes triggered by *Klebsiella pneumoniae* infection (days from 0 to 5), we were able to gather additional samples at day 9, 14, and 30. Surprisingly, microbial biofilm implanted in the peritoneum was eradicated almost immediately (Figure 1). Conversely, biofilm eradication on an implant sewn into the greater omentum occurred slower. The calculated number of bacteria on a withdrawn implant in the IO group increased at day 2, and biofilm eradication was only observed after the fifth day of the experiment. Therefore, we concluded that the greater omentum was capable of eradicating *Klebsiella pneumoniae* biofilm, although at a slower rate than the peritoneum.

**Figure 2:**
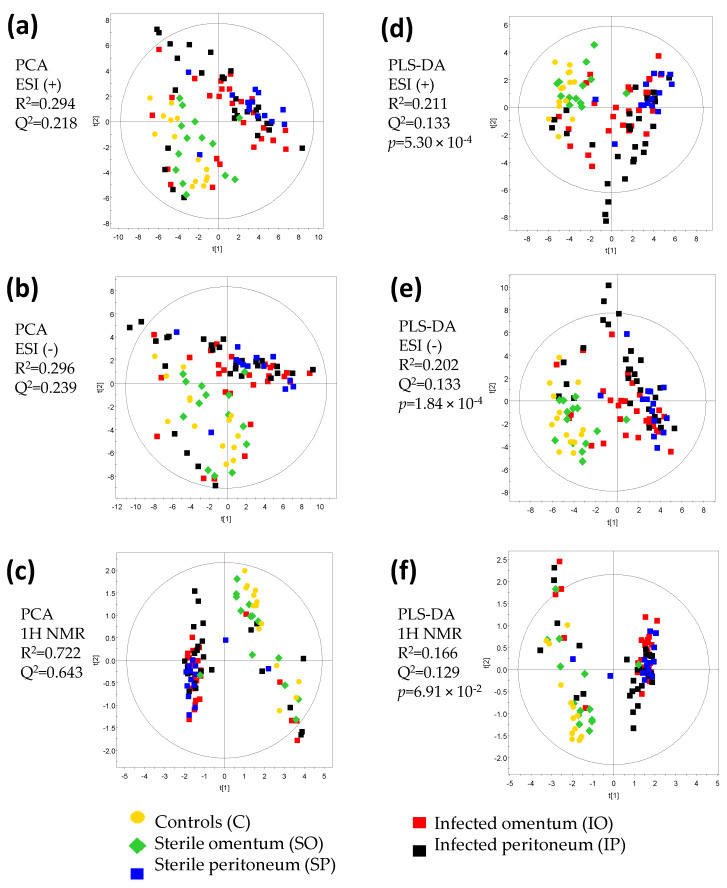
Principal component analysis (PCA) (**a–c**) models for all samples and day 0–5 obtained for each technique, and partial least squares discriminant analysis (PLS-DA) (**c,d**) models for all groups and days 0–5 obtained for each technique. (**a**,**d**) for positive electrospray ionization mode (ESI(+); (**b**) and (**e**) for negative electrospray ionization mode (ESI (-)); (**c**,**f**) for proton nuclear magnetic resonance (^1^H NMR). Models were obtained for the following number of variables: ESI(+)—319 variables, ESI(-)—382 variables, ^1^H NMR—22 variables. *R^2^* and *Q^2^* parameters are given for the two first components. *p*-value for ANOVA of the cross-validated residuals (CV ANOVA) is reported. Investigated groups are marked as follows: group IO (infected omentum)—red squares; IP (infected peritoneum)—black squares; SP (sterile peritoneum)—blue squares; SO (sterile omentum)—green diamonds; C (controls)—yellow dots.

Next, we analyzed urine samples using ^1^H NMR and LC–QTOF–MS operating in positive (ESI(+)) and negative (ESI(+)) electrospray ionization mode. Individual datasets obtained for each technique (^1^H NMR, ESI(+), and ESI(-)) were evaluated separately using principal component analysis (PCA) and partial least squares discriminant analysis (PLS-DA). Preliminary PCA analysis based on all groups of samples from all time points revealed a significant shift of the infected group towards controls throughout the course of recovery (Supplementary Figure S1). However, samples obtained after day 5 were scarce, and for all further statistical analysis, we focused on the initial effects of infection using only samples from days 0 to 5 (Figure 2). For each dataset, PCA (Figure 2a–c) and PLS-DA (Figure 2d–f) models showed very similar trends, but sample clustering was bolstered in PLS-DA models. These PLS-DA models, however, were used for data visualization purposes only, as their *R^2^* and *Q^2^* values were low, indicating lack of predictive power. This was probably due to the fact that some groups of samples showed very similar metabolic patterns, and could not be sufficiently discriminated in the PLS-DA model that was built using all studied groups of samples.

**Figure 3:**
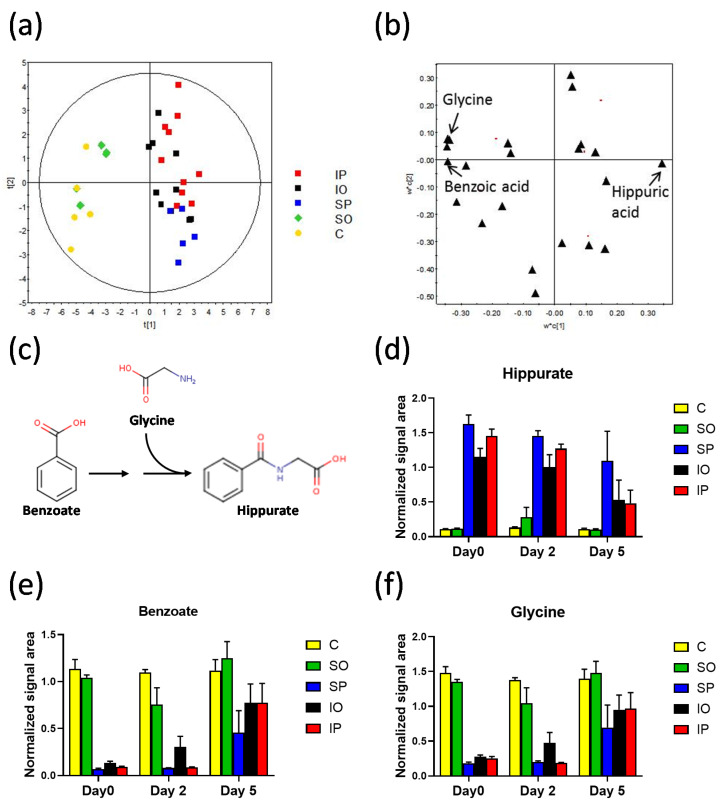
Plots for ^1^H NMR data (22 variables) of all samples from day 0 of the experiment. (**a**) Score plot of PLS-DA model, *R^2^* = 0.328, *Q^2^* = 0.206, CV ANOVA *p*-value = 0.16. (**b**) Loading plot for PLS-DA (**a**) with identification of selected variables. **(c)** Pathway of hippurate biosynthesis. Normalized signal intensities of (**d**) hippurate, (**e**) benzoate, and (**f**) glycine, quantified using ^1^H NMR spectroscopy. Data in panels (**d–f**) are presented as mean and standard error.

All three datasets based on rat urine metabolic profiles showed good separation between control rats (without surgical treatment) and both infected groups (IO and IP). Interestingly, PLS-DA models also revealed similarities between the control group and the sterile implant grafted in the greater omentum (SO). However, when the sterile implant was engrafted in the peritoneum (SP), the urine metabolic profile clustered together with groups that had a colonized implant (Figure 2d–f). As expected, metabolite levels changed significantly during the course of recovery, and the biggest effects were observed at the beginning of the experiment. Therefore, in order to investigate which metabolites were responsible for the observed grouping, we created a subset of PLS-DA models that were based only on day 0 samples (Figure 3a). This allowed us to select metabolites related to the early effects of surgery or infection. PLS-DA of ^1^H NMR data revealed that the metabolites responsible for clustering of groups into two regions were glycine, lactate, succinate, pyroglutamate, benzoate, allantoin, and hippurate (Figure 3b). Interestingly, three of these metabolites come from the hippurate biosynthesis pathway, namely, benzoate, glycine, and hippurate (Figure 3c). Indeed, the concentration of glycine was positively correlated with benzoate concentration and negatively with hippurate (Figure 3d–f). Levels of benzoate, glycine, and hippurate perfectly agreed with the results of PLS-DA analysis (Figure 3a). Hippurate is produced by bacteria, and thus its high abundance was expected in both infected groups (IO and IP); however, it was surprising to observe high levels of hippurate in the group with a sterile implant grafted in the peritoneum (SP). Differences related to hippurate pathway diminished over time (i.e., from day 0 to day 5), probably due to the ongoing process of biofilm eradication.

Although high levels of hippurate correlated strongly with the infection, it is important to note that hippurate is a natural metabolite that is commonly detected in the urine of animals and humans. Under standard physiological conditions, hippurate will be produced by the gut microbiome of plant-eating animals and is secreted in the urine. A potential explanation for high hippurate content in the SP group can be related to the recovery period after the engraftment of sterile implants in different locations in rats. It is possible that the level of discomfort was different between the animals with implants placed in the peritoneum or in the greater omentum. Such differences could have a behavioral impact and affect the feeding regime of rats. Furthermore, the surgical intervention in selected locations could affect intestinal homeostasis differentially and therefore contribute to changes in gut microbiome metabolic dynamics.

**Figure 4:**
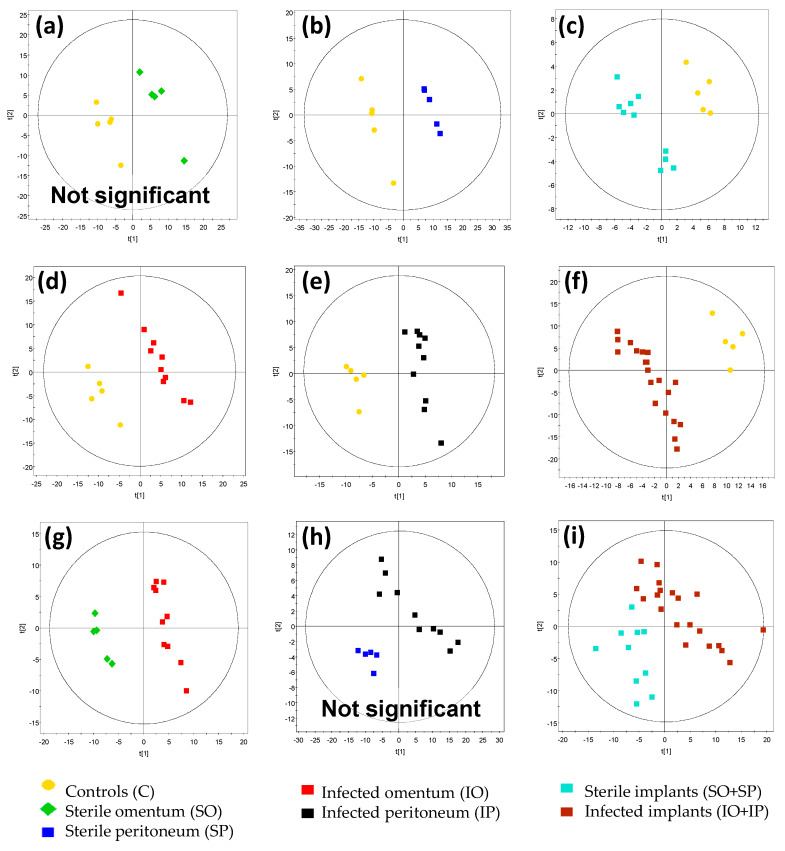
PLS-DA models for ESI(+) data (319 variables) of groups of samples from day 0 of the experiment: (**a**) sterile omentum vs. controls; (**b**) sterile peritoneum vs. controls; (**c**) sterile implants (omentum and peritoneum) vs. controls; (**d**) infected omentum vs. controls; (**e**) infected peritoneum vs. controls; (**f)** colonized implants (infected omentum and peritoneum) vs. controls; (**g**) infected omentum vs. sterile omentum; (**h**) infected peritoneum vs. sterile peritoneum; (**i**) colonized implants (infected omentum and peritoneum) vs. sterile implants (omentum and peritoneum). *R^2^* and *Q^2^* parameters are given for the two first components, and *p*-values for CV ANOVA for the presented models are found in Table 1.

Consequently, we moved to pairwise PLS-DA comparisons (Figure 4). The PLS-DA model comparing the SO group to C did not pass the validation tests (Table 1). This observation was in agreement with the pattern observed in the PCA score plots where these two groups clustered together, indicating that profiles of SO and C groups were similar. These data suggest that grafting the sterile implant into the greater omentum did not trigger a major metabolic response. Contrary, the SP vs. C model was statistically significant and suggested much greater metabolic change in the case of the grafting of the sterile implant into the peritoneum than into the greater omentum. This observation indicates a differential metabolic response depending on the implant location. 

Because the surgical procedure induced different metabolic responses in the case of the sterile implant when compared to the untreated rats, we moved to the comparison of sterile vs. colonized implants located either in the peritoneum or greater omentum. This approach allowed us to investigate the effect of infection independent of response to the surgery. In this case, only the IO vs. SO model was statistically significant, whereas the IP vs. SP showed no difference (Figure 4g,h; Table 1). These observations were unexpected, given the initial assumption about the greater omentum’s superior wound healing ability. However, a potential explanation for differential metabolic response to engraftment in the greater omentum and peritoneum lies in the different nature of the two locations. Unlike the peritoneum, the greater omentum is known for its inherent ability to cover foreign bodies in order to isolate them from the other organs located in the abdomen [32]. The encapsulation of a foreign body allows us to isolate the infection and treat it locally. On the other hand, infection in the peritoneum may generate a whole-body inflammatory response. This could result in significantly different dynamics in inflammatory response for the two investigated implant locations. In the case of the peritoneum, infection could trigger the inflammatory response to a much greater degree than when the implant was located in the greater omentum. Indeed, the unexpected rapid eradication of biofilm on colonized implants located in peritoneum was observed, suggesting that the inflammatory response must have been strong and rapid (Figure 1), whereas the greater omentum showed a slower rate of biofilm eradication. 

Finally, the IO vs. C and IP vs. C discriminatory models showed large changes between both “infected” groups versus controls without surgical treatment. However, in line with other analysis, the PLS-DA models set to discriminate colonized implant groups based on implantation site (IO vs. IP) did not reach statistical significance (Supplementary Figure S2). This observation suggests that the presence of *Klebsiella pneumoniae* biofilm results in similar metabolic perturbations, regardless of the implant location. 

In order to select the most discriminating metabolites, we used the variable importance in the projection (VIP) values > 1 (from PLS-DA models built for pair-wise comparisons separately for day 0, 2, and 5, separately) together with *p*-value < 0.05 (from Mann–Whitney test). Only metabolites meeting both criteria were considered significant. For significant data variables detected using LC–QTOF–MS, a target MS/MS analysis was performed for identification purposes (Supplementary Table S1). Certain metabolites were identified simultaneously in profiles from different techniques. Among others, citrate was found significant in ESI(+), ESI(-), and ^1^H NMR; betaine in ESI(+) and ^1^H NMR; hippurate, malate, taurine, allantoin, and indoxyl sulfate in ESI(-) and ^1^H NMR; and ferulate in ESI(+) and ESI(-). For further interpretation of metabolic changes, metabolites from one profile with the highest importance (lowest *p*-values) were chosen. The final set of significantly changing metabolites identified consisted of 62 metabolites: 14 significant metabolites identified by ESI(+), 30 metabolites by ESI(-), and 18 metabolites by ^1^H NMR.

**Figure 5:**
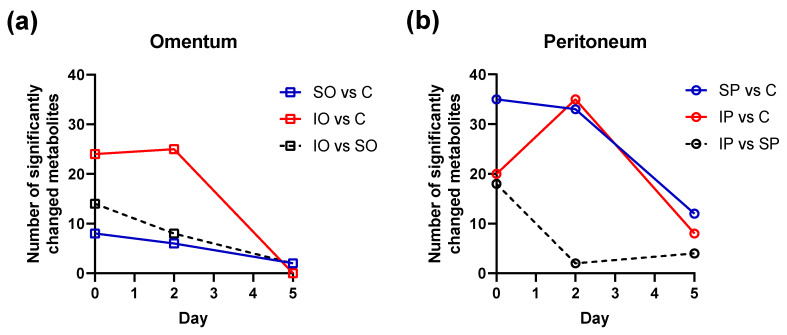
Number of significant metabolites (the variable importance in the projection values (VIP) > 1, *p*-value < 0.05) found in a merged dataset of ^1^H NMR- and LC–MS-identified features: (**a**) omentum, (**b**) peritoneum.

The most significant changes in all urine profiles appeared during the first two days of the experiment (Figure 5). Colonized implants placed in the omentum caused greater metabolic changes then sterile implants (Figure 5a). On the other hand, even sterile implants placed in the peritoneum caused large metabolic changes (in terms of number of significantly changed metabolites), similar to colonized implants (Figure 5b). 

For statistically significant metabolites, the percentage differences were determined (Table 2 and Supplementary Table S2). Metabolites associated with the tricarboxylic acid (TCA) cycle—citric acid, alpha-ketoglutaric acid, cis-aconitic acid, trans-aconitic acid, succinic acid, succinic acid semialdehyde, fumaric acid, and malic acid—were decreased significantly in all groups after surgery compared to controls (Supplementary Table S2). Lower values of TCA cycle intermediates depended on the type of the implant and the place of grafting. A greater and more significant decrease was observed in the case of the colonized implant located in the peritoneum. The same changes were observed in IO and IP vs. controls, but in this case, changes of relative percentage concentrations in both IO and IP groups compared to the C group were comparable (Supplementary Table S2). On the other hand, lactate was significantly reduced in all infected groups compared to controls without the surgery. However, when compared to the sterile implant controls, lactate was decreased in omentum (IO vs. SO) but increased in the peritoneum group (IP vs. SP) (Table 2). These findings may suggest changes related to TCA cycle flux and acetyl coenzyme A (acetyl-CoA) handling. Except for beta-oxidation, glycolytic flux and the TCA cycle oxidative turnover are the major sources of cellular ATP. During and after surgery, there is an increased need for energy and glucose, resulting in enhanced oxidative metabolism. Therefore, after depletion of glycogen by tissues, the liver upregulates gluconeogenesis to support recovery processes. Indeed, there have been reports of temporary insulin resistance as a metabolic response to surgery [34,35,36,37,38], and the severity of insulin resistance depends on magnitude of operation [39]. In our experiment, aforementioned changes related to TCA cycle metabolites were observed in all groups, but they were the least statistically important in the case of the SO group when compared to controls. This would suggest that surgical intervention in the greater omentum does not trigger hypermetabolism and inflammation mediators to a significant degree, which could be of importance when considering usage of the greater omentum for wound healing procedures. 

Post-surgical insulin resistance has been correlated with higher cortisol levels [40]. Elevated levels of other adrenocortical hormones excreted in the plasma and urine have been described [41,42]. In our study, we noted a significantly increased tetrahydrocortisone level at day 2 in SP and IP groups compared to controls, and its concentration was significantly higher in the IP group. Elevated levels were also observed in SO and IO groups, but without statistical significance. This could be evidence that metabolic changes in omentum groups were smaller than in peritoneum groups. Elevated levels of adrenocortical steroids in blood and urine have been linked with the changes in sodium, potassium, and nitrogen excretion [35,43]. Higher nitrogen excretion mainly in the form of urea is caused by intensified protein breakdown and using amino acids as an energy source directly (glutamine, alanine) or for gluconeogenesis. However, levels of urea were variable comparing to controls during first two days of the experiment, which could be related with different food intake between groups of animals.

In IO group compared to SO, choline levels were found to be significantly decreased. Changes in choline metabolites were also observed. Specifically, phosphorylcholine levels were elevated in all groups compared to controls, whereas tetrahydrofolic acid was decreased. Several studies reported a decrease of choline levels in plasma or serum after prolonged exercise, such as running a marathon [44,45,46], and after surgery, trauma, or stressful experiences in humans [47]. The drop of serum choline levels after abdominal surgery has been correlated with an increase in circulating cortisol, prolactin, adrenocorticotropic hormone (ACTH), and β-endorphin [48]. All these events are characterized by abnormal energy metabolism. It has been reported that choline and its products, including betaine, phosphatidylcholine, and acetylcholine, can either potentiate or attenuate responses of immune cells, and thus may have an immunomodulatory role [49]. Choline metabolites may influence an anti-inflammatory response of neutrophils and monocytes, as well as lymphocyte proliferation.

In course of the experiment, large differences in levels of three metabolites involved in hippurate biosynthetic pathway were observed. The increased concentration of hippurate correlated strongly with diminished pools of benzoate and glycine (Figure 3). Glycine and benzoic acid are combined in the process of hippurate biosynthesis (Figure 3c). The increase of hippuric acid level was positively correlated with elevated levels of isonicotynylglycine. Levels of hippurate are strongly dependent on diet as well as composition of gut microbiota. It was suggested that *Klebsiella pneumoniae* infection may change gut microbiome [50]. In a reported study, lower hippurate, citrate, lactate, choline, formate, and indoxyl sulfate levels were found, whereas creatinine was elevated [49]. Altered metabolic pathways were associated with post-operative reaction to stress and were also similar to the processes that occurs during inflammation. 

All groups that underwent the surgery showed reductions in levels of dietary metabolites (taurine, indolylacryloylglycine, kamlolenic acid, xylitol, stachyose), although not all of them were statistically significant (Supplementary Table S2). On the other hand, caffeic acid, ferulic acid, and their sulfate metabolites were significantly increased in the urine of rats after surgical engraftment of the sterile implant in the peritoneum, but did not change in the omentum group (Supplementary Table S2). Decrease in concentration of dietary metabolites could be associated with animals’ reduced food intake. From this perspective, our data suggest that the usage of the greater omentum may be advantageous for faster recovery after surgery.

Even on day 0 of the experiment, the profiles of IP and SP were very similar, showing only two differential metabolites—succinic acid and 4-hydroxylbenzaldehyde. The finding was in agreement with the number of bacteria left on the implant (Figure 1)—zero or less than 100 bacteria on the withdrawn implant from the IP group at day 2. 

The metabolic response in the urine of rats after grafting of the colonized implant was partially correlated with the histopathological description. Although in our previous report, engraftment of colonized implant induced severe histopathological changes in multiple organs (necrosis, ulcers) [51], we did not observe the same outcome in the animal subgroup used in our metabolomics experiment (Supplementary Table S3). In the animal cohort used for the metabolomics study, the observed histopathological changes were milder when compared to our previous report. However, the total number of pathological changes in pancreases was lower in the IO compared to the IP group. It is possible that when the colonized implant was placed in the peritoneum, the bacterial infection spread to a greater extent than in the case of the greater omentum. This would confirm the greater omentum’s ability for more effective control of the spread of inflammation in the body. 

Comparison between sterile implant groups (SO and SP) and rats that did not undergo surgical procedures (C) allowed us to assess the metabolic response to surgery specific to the implant location. When a sterile implant was introduced into the peritoneal cavity, observed changes were very similar to the organism’s reaction to a colonized implant, whereas in the case of sewing a sterile implant into the greater omentum, no significant differences were observed when compared to the control groups. Simultaneously, the similarity between the SP, IP, and IO groups was a major limitation of our study as it could be suggested that the bacterially colonized implant (IO) had the same metabolic effect as the sterile implant in the peritoneum (SP) group. In summary, our data are in agreement with the rationale of using the greater omentum in postoperative wound treatment, as the surgical intervention in the greater omentum did not significantly disturb metabolic homeostasis. 

## 3. Materials and Methods 

### 3.1. Design of the Experiment, Animal Handling and Sample Collection

Urine samples were collected at Wroclaw Medical University as a part of a bigger project. This experiment was conducted under the approval of the local bioethical committee of the Institute of Immunology and Therapy, Polish Academy of Science in Wroclaw (number of approval: 42/09). All animals used in the experiment were 10–14 weeks old Wistar female rats with an average body weight around 250 g. In total, 35 Wistar female rats were divided into two “treated” groups that underwent surgical intervention and implant grafting (*n* = 30) and a control group (C) (*n* = 5) that consisted of healthy rats without any surgical treatment. The control group received only an analgesic dose of pentobarbital at day 0 (at dose of 15 mg/kg of body mass). Surgically treated rats were divided into four groups and subjected to implantation of polypropylene surgical mesh. The implant was either sterile or covered with *Klebsiella pneumoniae* biofilm. 

For this purpose, *Klebsiella pneumoniae* K66 strain was chosen, due to its ability for effective biofilm formation on tested biomaterials, as shown in previous studies [51,52]. Bacterial density was estimated using a densitometer and serial dilutions in order to obtain a value of 3 × 10^8^ cells/mL. Next, a sterile fragment of implant was introduced to bacterial suspension and left for 24 h at 37 °C. After incubation, one group of biomaterials was used for implantation, whereas another was subjected to the procedure of biofilm de-attachment and cell counting. For the de-attachment procedure, the implants were introduced to 0.5 mL of mild detergent (0.5% saponine) and mixed for 1 minute. The de-attached bacterial cells were seeded into appropriate agar plates (McConkey Agar plate) and incubated for 24 h at 37 °C. After incubation, bacterial colonies were counted. The final number of biofilm-forming cells per implant was 5.2 × 10^6^ (±3.1 × 10^6^).

All animals were anesthetized intraperitoneally before surgery using pentobarbital (15 mg/kg of body mass). The skin of animals was disinfected with chlorhexidine solution in 10% ethanol. Additionally, 0.2% solution of lignocaine was used for local analgesia. Biomaterial, sterile or colonized, was left in the peritoneum (SP—sterile peritoneum, *n* = 5; IP—infected peritoneum, *n* = 10) or was grafted into the greater omentum (SO—sterile omentum, *n* = 5; IO—infected omentum, *n* = 10). Number of samples collected in each day of experiment and sample coding are summarized in Table 3. The healing process was carefully monitored. Rats were kept in metabolic cages that enabled urine collection. Urine was collected once a day between 8 and 10 a.m. Collected samples were representatives of 24 ± 2 h urine. The final volume of collected urine was not noted for all samples, and therefore it could not serve for normalization purposes. Urine was collected into containers with sodium azide to prevent metabolite degradation. Urine samples from the 2nd, 5th, 9th, 14th, and 30th days of the experiment were collected and divided into aliquots (for each technique), resulting in 137 urine samples in total. The experiment was stopped successively in the 2nd, 5th, 9th, 14th, and 30th days, and therefore the number of urine samples decreased with time of the experiment. Two rats from each of the infected groups (IO, IP) and one rat from groups SO, SP, and C were sacrificed on each time point, except day 0. As a result, seven rats were sacrificed per one time point. Table 3 presents the actual number of samples that were analyzed in this experiment. The number of urine samples in Table 3 is lower in some cases (day 5—IO, day 9—SO, day 14—SO) than what it should be, due to the fact that the urine sample was of insufficient quantity/quality for metabolomic profiling. Each rat was anesthetized with pentobarbital (15 mg/kg of body mass), and a lethal dose of ketamine was injected after withdrawal of implant and organs for histopathological analysis. Biomaterial was withdrawn and the number of bacteria left on implant surface was calculated using the same methodology as for counting the initial number of bacteria on the implant. Rats were also subjected to histopathological examination of internal organs and a place of implant grafting. All samples were stored at −70 °C until the day of analysis.

### 3.2. LC–QTOF–MS Analysis and Data Treatment

#### 3.2.1. Sample Preparation

Urine samples were thawed on ice and vortex. Then, protein precipitation and metabolite extraction was performed by mixing 50 µL of urine with 450 µL of cold (−20 °C) methanol. Samples were then vortexed for 1 min and left standing on ice for 5 min, centrifuged at 16,000 × *g* for 10 min at 4 °C, and then supernatant was filtered through a 0.22 μm nylon filter to a chromatographic vial with microinsert. Prepared samples were placed immediately in the thermostated autosampler at 4 °C to avoid metabolite degradation.

Due to a large number of samples and necessity of ion source cleaning, the whole set of samples was divided into three batches. Each batch contained 1/3 of randomly chosen representatives of each group. Quality control (QC) samples were prepared by pooling equal volumes of urine from each of the 46 samples from the first analytical batch. QC samples were independently prepared from this pooled urine for each of the three batches following the same procedure as for the rest of the samples. QC samples were analyzed at the beginning of the run and for every five samples throughout the run to provide a measurement of the system stability, instrument performance [53], and the reproducibility of the sample treatment procedure. 

#### 3.2.2. LC–QTOF Analysis

Samples were analyzed by an HPLC system (1260 Infinity series, Agilent Technologies, Waldbronn, Germany) consisting of a degasser, binary pump, and thermostated autosampler maintained at 4 °C connected to an Agilent Technologies QTOF (6530) mass spectrometer. Electrospray ionization (ESI) was used as an ion source. Samples (5 μL) were injected onto a reversed-phase column (Poroshell 120 EC-C18, 100 × 3.0 mm, 2.7um, Agilent) with a guard column thermostated at 40 °C. The system was operated in positive and negative mode at flow rate 0.5 mL/min with solvent A-water with 0.1% formic acid and solvent B-acetonitrile with 0.1% formic acid. Gradient started with 1% B, maintained 1 min, and then the gradient started from 1% B to 15% B at 6 min, then to 50% B at 8 min, 95% B at 10 min, reaching 100% B at 11 min and returned to starting conditions in 0.5 min, keeping the re-equilibration until 16 min. The detector operated in full scan mode from 50 to 1000 *m/z* for positive mode and from 50 to 1100 *m/z* for negative mode, with a scan rate of 1 scan per second. Accurate mass measurements were obtained by online mass correction to reference masses delivered continuously during analyses. Reference masses at *m/z* 121.0509 (protonated purine) and *m/z* 922.0098 (HP-921) were used in positive ion mode, whereas *m/z* 112.9856 (trifluoroacetic acid (TFA) anion) and *m/z* 1033.9881 (HP-0921) were applied in negative ion mode. The capillary voltage was set to 3000V for positive and 4000V in negative ionization mode, and the nebulizer gas flow rate was 10.5 L/min. Randomized samples were analyzed in two separate runs (first for positive and second for negative mode) in three batches.

#### 3.2.3. LC–QTOF Data Treatment

In order to generate a metabolic feature matrix (consisting of accurate masses, retention times, and signal abundances) an LC–QTOF molecular feature extraction (MFE) algorithm in Mass Hunter Qualitative Analysis B.05.00 software (Agilent Technologies) was used. Briefly, the MFE algorithm groups co-eluting ions corresponding to the same molecule including isotopes, charge state, adducts, and dimers. Parameters selected for data extraction were the same as those described previously [54]. The background noise cut-off was set to 500 counts, and allowed ions were [M+H]^+^, [M+Na]^+^, and [M+K]^+^ for positive ionization and [M–H]^-^ and [M+HCOO]^-^ for negative ionization. Neutral loss of water was allowed in both polarity modes. To align and filter data, we used Mass Profiler Professional (B.12.1, Agilent Technologies). Prior to the statistical analysis, we performed profound data filtration. Quality assurance procedure aimed to filter data on the basis of the information acquired across QCs, rejecting features present in less than 50% of QC samples and with coefficient of variation exceeding 30% in QCs. Further filtering was based on five studied groups, and features present in at least 90% of representatives in at least one group were kept. Before normalization, features annotated as metabolites of drugs used for surgical procedures were discarded from the dataset. LC–QTOF analysis followed by the quality assurance procedure led to the selection of 319 features for positive ion mode ESI(+) and 382 for negative ion mode ESI(-), which were measured in a reproducible way. Generated sets of data for positive and negative modes were normalized in MATLAB by means of PQN (probabilistic quotient normalization) [55], using sample median as a reference, and logarithmically transformed to approximate normal distribution (MS Excel (Microsoft)).

### 3.3. ^1^H NMR Analysis and Signal Assignment

Urine samples were thawed and vortexed. A total of 570 μL of each urine sample was mixed with 30 μL of KF solution and centrifuged 10 min at 12,000 × *g*. A total of 540 μL of supernatant was transferred to a new tube. Then, 30 μL of phosphate-buffered saline (PBS) was added in order to keep a stable pH and therefore stabilize a chemical shift in the NMR spectrum. PBS (pH = 7.00) contained 30% of deuterated water (D_2_O) for deuterium lock and 3 mM sodium salt of trimethylsilyl-2,2,3,3-tetradeuteropropionic acid (TSP) as internal standard. Next, samples were vortexed, and 550 μL was transferred into a 5 mm NMR tube and measured with a NMR spectrometer. All spectra were recorded by a Bruker Avance II 600MHz NMR spectrometer (Bruker BioSpin Ltd., Milton, ON, Canada). One-dimensional proton ^1^H NMR analysis was carried out using the NOESY pulse sequence with water suppression. Each sample was left inside the spectrometer for 2–3 min in order to reach stable temperature. All measurements were performed in identical conditions: relaxation delay: 3.5s, number of scans: 128, number of points: 32,768, acquisition time: 2.379 s, mixing time: 100 ms. The resulting spectra were manually processed (phase and baseline correction, referencing the TSP peak. Region of solvent signal (4.70–4.92 ppm) was removed. Signals were aligned using correlation optimized warping (COW) algorithms and icoshift [56]. Data were normalized using PQN (probabilistic quotient normalization), and data metabolites were identified as previously published [57] and using the Human Metabolome Database (HMDB). For metabolite quantification, only metabolite resonances without overlap were used. Signals were integrated using an in-house MATLAB algorithm leading to the set of 22 identified metabolites used for further analysis. 

### 3.4. Statistical Analysis

Three normalized and logarithmically transformed data matrixes (^1^H NMR, ESI(+), and ESI (-)) were analyzed separately in SIMCA P+12 (Umetrics). For PCA (principal component analysis) and PLS-DA (partial least squares discriminant analysis), Pareto scaling was used. Statistical models were built for each pair of studied cases according to different days of sample collection. PLS-DA models were validated using CV ANOVA, and models that the passed validation test (CV ANOVA, *p* < 0.05) were used to select discriminant variables [58]. Significance of variables was assessed by variance of importance to the model VIP > 1. Differences in metabolite concentrations were also evaluated by Mann–Whitney *U* test (*p* ≤ 0.05) calculated using STATISTICA 13 (StatSoft, Cracow, Poland). Finally, for metabolites that appeared as significant in at least one of the comparisons of the studied groups, percentage differences in relative concentration metabolites were calculated on the basis of signal areas for each pair of compared groups. Tables with NMR and LC-MS quantified signals are provided in the supplementary materials (Supplementary Tables S4–S6).

### 3.5. Metabolites Identification

Metabolic features that were found to be significant in multivariate analysis and passed the Mann–Whitney *U* test were chosen for additional MS/MS analysis. Corresponding ions (for positive and negative mode) were targeted for collision-induced dissociation (CID) fragmentation on the basis of previously determined accurate mass and retention time. The MS/MS analyses were performed under identical chromatographic conditions to the primary analysis. Accurate mass and isotopic pattern distribution were studied, and using the GMF (Generate Molecular Formula) algorithm (Mass Hunter Qualitative Analysis, Agilent, Santa Clara, CA, USA), we generated a probable formula of a compound. The accurate masses for the precursor and product ions obtained in LC–QTOF–MS/MS analysis were studied and compared to the spectra of reference compounds found in online accurate mass databases (Metlin: (www.metlin.scripps.edu), HMDB: (www.hmdb.ca)). When there was a positive match of the spectra in the library and MS/MS spectra were obtained, we annotated the compound (level 2 in the confidence level scale according to Sumner L. W. et al. (2007) [59]). For the compounds that we were not able obtain clear MS/MS spectra (in case of very narrow and small peaks), we made the annotation only on the basis of the monoisotopic mass and isotopic pattern distribution (confidence level 3 in the confidence level scale according to Sumner L. W. et al. (2007) [59]) ( Supplementary Table S1).

### 3.6. Quantitative Analysis

Quantitative measurements in non-targeted metabolomics are relative changes based on the intensities of signals (abundances of detected ions) and, in fact, they are at best semi-quantitative. Percentage of change was calculated on the basis of the difference of the average of the signal’s abundance of a selected metabolite between studied groups divided by the average of the signal’s abundance in the second group. The calculated changes are summarized in Table 2 and Supplementary Table S2.

## 4. Conclusions

We found that the grafting of implants covered with *Klebsiella pneumoniae* biofilm, either to the greater omentum or peritoneum, triggered in both cases metabolic changes related to surgical stress, similar to those observed during inflammation. The observed changes were more evident when the colonized implant was placed in the peritoneum. Results confirmed germicidal abilities of greater omentum. Bacterial biofilm on grafted implant was eradicated. Moreover, biofilm eradication was also observed on implants grafted to the peritoneum. Our studies proved the abilities of the greater omentum to eradicate bacterial biofilm of a grafted implant. Understanding the benefits coming from using the greater omentum for surgical purposes will contribute to possibilities of future applications of the omentum in preventing infection development and healing wounds with high inflammation risk, especially those associated with bacterial biofilm formation. Finally, we showed that activation of the greater omentum for biofilm eradication perturbs the metabolism to a much lesser degree than in the case of the peritoneum, allowing for better maintenance of metabolic homeostasis and improved recovery in rats. Our findings should contribute to the increase of practical use of omental flap in clinical procedures of wound healing and tissue regeneration in the future. 

## Figures and Tables

**Table 1:** Parameters of PLS-DA models presented in Figure 4 obtained for ESI(+) data. *R^2^* and *Q^2^* parameters for the two first components. *p*-values are given for CV ANOVA.

Figure	Comparison	*R^2^* Value	*Q^2^* Value	CV ANOVA *p*-Value	Model Significance
Figure 4a	SO vs. C	0.976	0.804	2.40 × 10^−1^	No
Figure 4b	SP vs. C	0.999	0.93	1.20 × 10^−2^	**Yes**
Figure 4c	SO + SP vs. C	0.955	0.739	1.17 × 10^−2^	**Yes**
Figure 4d	IO vs. C	0.984	0.903	9.04 × 10^−4^	**Yes**
Figure 4e	IP vs. C	0.974	0.792	1.69 × 10^−3^	**Yes**
Figure 4f	IO + IP vs. C	0.963	0.913	6.44 × 10^−9^	**Yes**
Figure 4g	IO vs. SO	0.975	0.773	1.84 × 10^−2^	**Yes**
Figure 4h	IP vs. SP	0.954	0.354	3.57 × 10^−1^	No
Figure 4i	IO + IP vs. SO + SP	0.790	0.382	3.50 × 10^−2^	**Yes**

**Table 2:** List of identified metabolites significantly different between the studied groups of infected omentum versus sterile omentum (IO vs. SO), infected peritoneum versus sterile peritoneum (IP vs. SP), infected omentum versus infected peritoneum (IO vs. IP), and sterile omentum versus sterile peritoneum (SO vs. SP).

Day	d0		d2		d5		d0		d2		d5		d0		d2		d5		d0		d2		d5	
Groups	IO_SO		IO_SO		IO_SO		IP_SP		IP_SP		IP_SP		IO_IP		IO_IP		IO_IP		SO_SP		SO_SP		SO_SP	
**TCA CYCLE, CoA Biosynthesis**																								
N-acetylaspartate *(p, a)*	5%	-	33%	-	−33%	-	12%	-	7%	-	18%	-	−22%	-	−12%	-	−14%	-	−17%	-	−30%	-	52%	-
L−Lactic acid *(m)*	−88%	**	−83%	-	138%	-	89%	*	30%	-	98%	-	−12%	-	302%	-	16%	-	1259%	**	1245%	**	209%	-
Formic acid *(m)*	−38%	-	−83%	**	−79%	-	−63%	**	−22%	-	166%	-	13%	-	−5%	-	35%	-	−55%	-	36%	-	325%	*
Acetic acid *(m)*	−82%	**	−93%	-	−62%	-	18%	*	−7%	-	271%	-	11%	-	208%	-	40%	-	575%	**	424%	*	569%	*
Pyruvic acid *(m)*	−15%	*	−38%	-	−14%	-	8%	-	2%	-	42%	-	15%	-	26%	*	58%	-	62%	**	66%	*	70%	*
Citric acid *(m)*	14%	-	474%	-	313%	-	1%	-	1%	-	−36%	-	20%	-	18%	-	4%	-	36%	*	30%	-	−77%	*
alpha-ketoglutaric acid *(m)*	60%	-	68%	-	53%	-	8%	-	19%	-	−2%	-	45%	-	52%	-	84%	*	76%	**	60%	-	19%	-
cis-Aconitic acid *(m)*	7%	-	6%	-	159%	-	−23%	-	4%	-	−7%	-	26%	-	6%	-	30%	-	−19%	-	−3%	-	12%	-
trans-Aconitic acid *(m)*	18%	*	33%	-	−27%	-	8%	-	−3%	-	7%	-	0%	-	3%	-	−7%	-	−15%	*	−10%	-	−3%	-
Succinic acid *(m)*	−38%	-	−66%	-	−16%	-	−24%	-	−9%	-	27%	-	52%	*	50%	*	136%	*	65%	-	61%	-	263%	*
Succinic acid semialdehyde *(n, a)*	−20%	-	−10%	-	178%	*	69%	-	45%	-	10%	-	21%	-	43%	-	81%	-	155%	*	128%	-	−29%	-
Fumaric acid *(m)*	159%	-	331%	-	387%	-	−14%	-	−6%	-	−27%	-	40%	-	57%	-	−23%	-	−39%	-	−22%	-	−69%	*
Malic acid *(n, a)*	42%	-	64%	*	25%	-	−2%	-	2%	-	−29%	-	15%	-	58%	*	53%	-	−21%	-	−2%	-	−12%	-
																								
**AMINO ACIDS**																								
Pyroglutamic acid *(n, b)*	−11%	-	38%	-	21%	-	4%	-	−10%	-	−13%	-	−26%	-	14%	-	51%	-	−14%	-	−25%	-	9%	-
L-Alanine *(m)*	−49%	**	−89%	-	−63%	**	−9%	-	9%	-	138%	-	8%	-	40%	-	0%	-	59%	*	152%	*	514%	*
																								
****TRYPTOPHAN METABOLISM****																								
Tryptophyl-Glutamate *(n, b)*	14%	-	−12%	-	−22%	-	−3%	-	29%	-	−4%	-	46%	-	31%	-	42%	-	25%	-	92%	*	75%	-
Indoxyl sulfate *(n, a)*	−18%	-	−17%	-	5%	-	−30%	-	68%	-	1%	-	18%	-	−16%	-	37%	-	1%	-	71%	-	33%	-
																								
**HIPPURATE BIOSYNTHESIS**																								
Benzoic acid *(m)*	−83%	**	−76%	-	−21%	-	33%	-	8%	-	70%	-	44%	-	258%	*	0%	-	1419%	**	857%	**	173%	*
Glycine *(m)*	−74%	**	−68%	-	38%	-	39%	*	−7%	-	39%	-	8%	-	155%	-	−2%	-	639%	**	426%	*	113%	-
Hippuric acid *(m)*	310%	**	877%	-	442%	-	−11%	-	−13%	-	−56%	-	−21%	-	−21%	-	10%	-	−93%	**	−81%	**	−91%	*
																								
**CHOLINE, LIPID METABOLISM**																								
Choline *(p, a)*	−8%	-	−67%	*	−7%	-	54%	-	4%	-	228%	-	45%	-	−24%	*	22%	-	145%	*	139%	*	333%	-
Betaine *(m)*	7%	-	−34%	-	−19%	-	−18%	-	−8%	-	−5%	-	15%	-	−4%	-	46%	-	21%	-	24%	*	5%	-
Creatinine *(p, a)*	48%	-	5%	-	82%	-	4%	-	−20%	-	8%	-	−3%	-	18%	-	−16%	-	−32%	-	−11%	-	−50%	*
Phosphorylcholine *(p, b)*	52%	-	36%	-	243%	-	−32%	-	−27%	-	−49%	-	2%	-	14%	-	27%	-	−55%	-	−39%	-	−81%	-
Tetrahydrofolic acid *(n, b)*	1%	-	1%	-	66%	-	139%	*	−4%	-	10%	-	−34%	-	−9%	-	48%	-	56%	-	−14%	-	−2%	-
																								
**ACYL GLYCINES**																								
Isonicotinylglycine *(p, a)*	126%	**	75%	-	na	-	−41%	*	−16%	-	−54%	-	33%	-	8%	-	−51%	-	−65%	*	−48%	-	na	-
Phenylacetylglycine *(p, a)*	55%	-	38%	-	1041%	-	−35%	-	40%	-	−41%	-	17%	-	−23%	-	−1%	-	−51%	-	−22%	-	−95%	*
3-Hydroxyhippuric acid *(n, a)*	78%	-	129%	*	683%	-	12%	-	−1%	-	65%	-	−2%	-	4%	*	21%	-	−39%	-	−55%	-	−74%	-
																								
**HORMONES**																								
Tetrahydrocortisone *(p, b)*	16%	-	18%	-	−8%	-	33%	-	44%	*	−7%	-	−18%	-	−13%	-	−4%	-	−6%	-	6%	-	−2%	-
Dihydrocortisol *(p, b)*	56%	-	34%	-	−47%	-	5%	-	20%	-	−46%	-	9%	-	−18%	-	−6%	-	−27%	-	−27%	-	−4%	-
																								
**DIETARY METABOLITES**																								
Taurine *(p, a)*	12%	-	−23%	-	80%	-	−30%	-	26%	-	−12%	-	−1%	-	−24%	-	−22%	-	−38%	-	24%	-	−62%	*
Indolylacryloylglycine *(n, a)*	-4%	-	7%	-	103%	-	−18%	-	−17%	-	−10%	-	46%	-	41%	-	36%	-	25%	-	9%	-	−40%	-
kamlolenic acid *(p, b)*	143%	-	−8%	-	25%	-	35%	-	34%	-	160%	-	19%	-	7%	-	−4%	-	−34%	-	56%	-	99%	-
Xylitol (+Ribitol) *(n, a)*	16%	-	−1%	-	73%	-	15%	-	35%	-	5%	-	−1%	-	−19%	-	11%	-	−2%	-	11%	-	−32%	-
Stachyose *(n, a)*	13%	-	−40%	*	−61%	-	648%	-	17%	-	−1%	-	−58%	-	−27%	*	−32%	-	176%	*	42%	-	73%	-
																								
**POLYPHENOLS INTAKE**																								
Phenol sulphate *(n, a)*	10%	-	−8%	-	60%	-	−46%	*	6%	-	−52%	*	34%	-	0%	-	11%	-	−34%	-	16%	-	−66%	*
Tyrosol 4-sulfate *(n, b)*	5%	-	−15%	-	−12%	-	−10%	-	−32%	-	−40%	-	−17%	-	12%	-	73%	-	−28%	-	−11%	-	17%	-
Pyrocatechol sulphate *(n, a)*	−5%	-	37%	*	−14%	-	−36%	**	−8%	-	−14%	-	2%	-	0%	*	−11%	-	−31%	*	−33%	*	−11%	-
																								
**URIC ACID**																								
Allantoin *(n, a)*	29%	-	5%	-	58%	-	45%	*	12%	-	−20%	-	5%	-	1%	-	14%	-	19%	-	8%	-	−42%	-
																								
**CAFFEIC ACID FERRULIC ACID**																								
Caffeic acid 3-sulfate / Caffeic acid 4-sulfate *(p, a)*	−13%	-	−1%	-	−15%	-	−24%	*	−15%	-	36%	-	10%	-	30%	-	5%	-	−4%	-	11%	-	69%	-
Dihydrocaffeic acid 3-sulfate *(n, a)*	56%	-	51%	-	−4%	-	−35%	*	−24%	-	−44%	*	15%	-	4%	-	22%	-	−52%	*	−48%	*	−28%	-
Ferulic acid *(n, a)*	71%	*	19%	-	−4%	-	−40%	**	−4%	-	−34%	-	0%	-	0%	-	35%	-	−65%	*	−20%	-	−7%	-
Ferulic acid 4-O-sulfate *(n, b)*	39%	-	31%	-	0%	-	−44%	*	−24%	-	−35%	-	−10%	-	28%	-	29%	-	−64%	*	−26%	-	−17%	-
Dihydroferulic acid *(n, a)*	−54%	-	60%	-	56%	-	56%	-	6%	-	−27%	-	9%	-	480%	-	134%	-	270%	-	284%	*	10%	-
Ferrulic acid 4-O-glucuronide *(n, a)*	175%	**	31%	-	159%	-	−18%	-	10%	-	−75%	-	17%	-	4%	-	41%	-	−65%	-	−13%	-	−86%	-
Dihydrocaffeic acid 3-O-glucuronide *(n, b)*	88%	-	34%	-	8274%	-	−27%	-	−26%	*	−31%	-	21%	-	−27%	-	0%	-	−52%	*	−60%	*	−99%	-
Dihydroferulic acid 4-O-glucuronide *(n, b)*	96%	-	60%	-	642%	-	−27%	*	−11%	-	−41%	-	21%	-	−14%	-	90%	-	−55%	*	−52%	*	−85%	-
Dihydroferuloylglycine *(p, b)*	229%	-	−5%	-	144%	-	−48%	-	5%	-	−29%	-	57%	-	29%	-	130%	-	−75%	*	43%	-	−33%	-
																								
**OTHER METABOLITES**																								
Urea *(m)*	15%	-	−14%	-	3065%	-	−25%	*	7%	-	−28%	-	10%	-	−7%	-	53%	-	−32%	**	−12%	-	15%	-
Histamine *(p, a)*	36%	-	27%	-	52%	-	−30%	-	24%	-	−39%	*	16%	-	9%	-	13%	-	−40%	-	7%	-	−55%	-
17,21-Dihydroxypregnenolone *(p, b)*	78%	-	91%	-	−3%	-	−9%	-	121%	-	11%	-	−7%	-	−24%	-	−25%	-	−53%	-	−13%	-	−14%	-
Isohomovanillic acid *(n, a)*	11%	-	30%	-	−18%	-	−14%	-	18%	-	−20%	-	15%	-	79%	-	31%	-	−11%	-	63%	-	28%	-
4-Hydroxybenzaldehyde *(n, a)*	−30%	-	0%	-	−20%	-	−21%	-	21%	-	−16%	-	44%	*	15%	-	−11%	-	62%	-	39%	-	−7%	-
3-Hydroxydodecanedioic acid *(n, b)*	−6%	-	−27%	-	−35%	-	−3%	-	1%	-	−2%	-	47%	-	25%	-	24%	-	51%	*	73%	-	86%	*
3-Hydroxyisoheptanoic acid / Ethyl 2-hydroxyisovalerate *(n, b)*	15%	-	−13%	-	−14%	-	66%	-	51%	-	61%	-	−12%	-	24%	-	2%	-	27%	-	116%	*	90%	-
O-methoxycatechol-O-sulphate *(n, b)*	10%	-	7%	-	−17%	-	−47%	**	10%	-	−50%	-	18%	-	−13%	-	−5%	-	−43%	*	−11%	-	−42%	-
Trigonelline *(m)*	30%	*	−9%	-	8%	-	−16%	-	−22%	-	−11%	-	0%	-	8%	-	10%	-	−34%	**	−29%	**	0%	-
Oxolan-3-one *(n, a)*	35%	*	20%	*	3%	-	−10%	-	9%	-	−6%	-	11%	-	8%	*	10%	-	−26%	*	−2%	-	1%	-
N-Acetyl-7-O-acetylneuraminic acid *(n, a)*	100%	-	−49%	-	7%	-	−8%	-	−1%	-	46%	-	136%	-	−21%	-	33%	-	9%	-	53%	-	81%	-
Ethanol *(m)*	−2%	-	−32%	-	−9%	-	19%	-	3%	-	65%	-	−10%	-	2%	-	38%	-	3%	-	1%	-	42%	*
2-Octenedioic acid / cis-4-Octenedioic acid / trans-3-Octenedioic acid *(n, b)*	−7%	-	40%	-	74%	-	152%	*	19%	-	24%	-	−17%	-	2%	-	44%	-	126%	*	−14%	-	2%	-
beta-D-Mannosylphosphodecaprenol *(n, b)*	−68%	**	−45%	*	−5%	-	141%	*	37%	-	101%	*	−41%	-	22%	*	−11%	-	345%	*	202%	*	89%	-

Percentage change between the two groups was calculated using following equation: 100% * (group 1-group2)/group 2, *p*-values: * *p*-value < 0.05; ** *p*-value < 0.01; - *p*-value not significant. (n) metabolites found in negative polarity mode; (p) metabolites found in positive polarity mode; (m) metabolites found in ^1^H NMR; (a) metabolites annotated by MS/MS spectra and MS fragmentation pattern; (b) metabolites annotated putatively by exact mass data and isotopic pattern distribution. Color coding: red- increase in the first comparing to the second group, light blue-decrease in the first comparing to the second, yellow – denotes statistically significant change.

**Table 3:** Experiment group coding and number of samples in each group depending on the day of the experiment. Only samples from day 0, 2, and 5 were used for statistical analysis.

				Number of Urine Samples					
Implant	Place of Implant Grafting	Name of the Group	Initial Number of Rats	Day 0	Day 2	Day 5	Day 9	Day 14	Day 30
Sterile	Peritoneum	SP	5	5	5	5	3	2	1
Sterile	Omentum	SO	5	5	5	4	2	1	1
Colonized	Peritoneum	IP	10	10	10	8	6	4	2
Colonized	Omentum	IO	10	10	10	7	6	4	2
-	No surgery	C	5	5	5	4	3	2	1

## Supplementary Materials

The following are available online at https://www.mdpi.com/2076-0817/9/5/399/s1. Figure S1. PCA models for all samples for all days obtained for (**a**) ESI(+) and (**b**) ESI(-). Figure S2. PLS-DA models for group IO vs. IP day 0 obtained for (**a**) ESI(+) 319 variables, (**b**) ESI(-) 382 variables, and (**c**) ^1^H NMR 22 variables. Table S1. Identification list of metabolites annotated by LC–QTOF–MS. Table S2. List of annotated metabolites significantly different between the studied groups sterile omentum versus controls (SO vs. C), sterile peritoneum versus controls (SP vs. C), infected omentum versus controls (IO vs. C), infected peritoneum versus controls (IP vs. C). Table S3. Results of histopathological examination of the animals included in the study. Table S4. ^1^H NMR quantified and normalized signals used for statistical analysis. Table S5. LC-QTOF-MS ESI(+) quantified and normalized signals used for statistical analysis. Table S6. LC-QTOF-MS ESI(-) quantified and normalized signals used for statistical analysis.
