# Supplementary material for: LC-QTOF-MS and 1H NMR Metabolomics Verifies Potential Use of Greater Omentum for Klebsiella pneumoniae Biofilm Eradication in Rats
Source: Pathogens. 2020 May 21;9(5):399. doi: 10.3390/pathogens9050399 (PMC7281169; doi:10.3390/pathogens9050399)
Supplement: Supplementary file 1 [file pathogens-09-00399-s001.zip › Supplementary data PROOF/Table S1_PROOF.docx]

**Table S1.** Identification list of metabolites annotated by LC-QTOF-MS.

| **Metabolite /Analyte name** | **Measured mass (Da)** | **RT (min)** | **Calculated mass error [ppm]** | **Ionization mode** | **Level of identification** | **MS/MS fragments used for identification (or standard if one was used).** | **Name of spectral database used for identification** |
| --- | --- | --- | --- | --- | --- | --- | --- |
| Oxolan-3-one (n, a) | **86.0373** | **1.3** | 6 | **ESI(-)** | a | 85.0299 + isotopic pattern distribution | HMDB |
| Succinic acid semialdehyde (n, a) | **102.0319** | **1.1** | 2 | **ESI(-)** | a | 57.0361, 59.0152, 101.0247 | HMDB |
| Choline (p, a) | **103.0996** | **1.0** | -1 | **ESI(+)** | a | 58.0673, 60.0841, 104.1067 | Metlin |
| Histamine (p, a) | **111.0797** | **0.9** | 0 | **ESI(+)** | a | 41.0383, 55.9731, 68.0143, 68.0454, 83.0577, 95.0575 | Metlin |
| Creatinine (p, a) | **113.0584** | **1.1** | -5 | **ESI(+)** | a | 44.0508, 59.0739, 70.0658, 86.0715 (+standard) | Metlin |
| 4-Hydroxybenzaldehyde (n, a) | **122.0368** | **8.8** | 0 | **ESI(-)** | a | 91.0185, 92.0259, 93.0339, 121.0296 | Metlin |
| Taurine (p, a) | **125.0142** | **1.0** | -4 | **ESI(+)** | a | 108.0113, 126.0220 | Metlin |
| Pyroglutamic acid (n, b) | **129.0423** | **1.3** | -2 | **ESI(-)** | b | monoisotopic mass + isotopic pattern distrubution | - |
| Malic acid (n, a) | **134.0213** | **1.1** | -2 | **ESI(-)** | a | 59.0144, 71.0149, 89.0255, 115.0036, 133.0138 (+standard) | Metlin |
| 3-Hydroxyisoheptanoic acid / Ethyl 2-hydroxyisovalerate (n, b) | **146.0940** | **9.9** | -2 | **ESI(-)** | b | monoisotopic mass + isotopic pattern distrubution | - |
| Xylitol (n, a) | **152.0679** | **1.0** | -4 | **ESI(-)** | a | 59.0146, 71.0138, 78.0265?, 89.0265, 101.0232, 119.0314, 137.0357 | HMDB |
| Allantoin (n, a) | **158.0436** | **1.0** | -2 | **ESI(-)** | a | 59.0266, 71.0260, 97.0048, 114.0308, 140.0085, 157.0364 | Metlin |
| Phosphorylcholine (p,b) | **169.0507** | **9.7** | 2 | **ESI(+)** | b | monoisotopic mass + isotopic pattern distrubution | - |
| 2-Octenedioic acid / cis-4-Octenedioic acid / trans-3-Octenedioic acid (n, a) | **172.0732** | **9.1** | -2 | **ESI(-)** | a | 59.0152, 109.0645, 127.0756, 153.0571, 171.0657 | HMDB |
| Phenol sulphate (n, a) | **173.9982** | **6.4** | -3 | **ESI(-)** | a | 65.0412, 93.0348, 172.9915 | HMDB |
| N-acetylaspartate (p, a) | **175.0475** | **1.2** | -3 | **ESI(+)** | a | 88.0410, 116.0443, 134.0462, 159.0530, 176.0669 | HMDB |
| Isonicotinylglycine (p, a) | **180.0504** | **7.1** | -17 | **ESI(+)** | a | 135.0546, 163.0482, 181.0959 | HMDB |
| Isohomovanillic acid (n, a) | **182.0574** | **8.7** | -3 | **ESI(-)** | a | 94.0369, 122.0315, 137.0590 | Metlin |
| Pyrocatechol sulphate (n, a) | **189.9930** | **5.5** | -3 | **ESI(-)** | a | 109.0294; 188.9858; 79.958 | HMDB |
| Phenylacetylglycine (p, a) | **193.0721** | **8.9** | -9 | **ESI(+)** | a | 30.0319, 65.0363, 76.0360, 91.0505, 120.0764, 148.0723, 176.0653 | Metlin |
| ferulic acid (n, a) | **194.0575** | **8.3** | -2 | **ESI(-)** | a | 97.0667, 123.0313, 134.0364, 149.0597, 159.0626, 178.0275 | HMDB |
| 3-Hydroxyhippuric acid (n, a) | **195.0525** | **6.7** | -3 | **ESI(-)** | a | 93.0361, 150.0566 | Metlin |
| Dihydroferulic acid (n, a) | **196.0730** | **8.9** | -3 | **ESI(-)** | a | 59.0153, 123.0449, 135.0471, 151.0746, 177.0545 | HMDB |
| O-methoxycatechol-O-sulphate (n, b) | **204.0086** | **7.4** | -3 | **ESI(-)** | b | monoisotopic mass + isotopic pattern distrubution | - |
| Indoxyl sulfate (n, a) | **213.0090** | **7.7** | -3 | **ESI(-)** | a | 79.9579, 80.9657, 104.0513, 132.0448 | Metlin, HMDB |
| Tyrosol 4-sulfate (n, b) | **218.0246** | **9.7** | -1 | **ESI(-)** | b | monoisotopic mass + isotopic pattern distrubution | - |
| Indolylacryloylglycine (n, a) | **244.0842** | **9.7** | -2 | **ESI(-)** | a | 74.0252, 100.0038, 114.0948, 142.0657, 168.0450, 199.0887, 225.1109 | HMDB |
| 3-Hydroxydodecanedioic acid (n, b) | **246.1461** | **9.6** | -3 | **ESI(-)** | b | monoisotopic mass + isotopic pattern distrubution | - |
| Dihydroferuloylglycine (p, b) | **253.0937** | **8.2** | -5 | **ESI(+)** | b | monoisotopic mass + isotopic pattern distrubution | - |
| Caffeic acid 3-sulfate / Caffeic acid 4-sulfate (p, a) | **259.9979** | **0.8** | -5 | **ESI(+)** | b | monoisotopic mass + isotopic pattern distrubution | - |
| Dihydrocaffeic acid 3-sulfate (n, a) | **262.0138** | **6.5** | -4 | **ESI(-)** | a | 95.0505, 137.0601, 181.0496, 217.016 | HMDB |
| Ferulic acid 4-O-sulfate (n, b) | **274.0138** | **8.3** | -3 | **ESI(-)** | b | monoisotopic mass + isotopic pattern distrubution | - |
| kamlolenic acid (p, b) | **294.2179** | **10.5** | -5 | **ESI(+)** | b | monoisotopic mass + isotopic pattern distrubution | - |
| Tryptophyl-Glutamate (n, b) | **333.1233** | **10.1** | -28 | **ESI(-)** | b | monoisotopic mass + isotopic pattern distrubution | - |
| 17,21-Dihydroxypregnenolone (p, b) | **348.2283** | **9.7** | -5 | **ESI(+)** | b | monoisotopic mass + isotopic pattern distrubution | - |
| N-Acetyl-7-O-acetylneuraminic acid (n, a) | **351.1159** | **1.3** | -2 | **ESI(-)** | a | 87.0092 + isotopic pattern distribution | HMDB |
| Dihydrocaffeic acid 3-O-glucuronide (n, b) | **358.0884** | **6.1** | -4 | **ESI(-)** | b | monoisotopic mass + isotopic pattern distrubution | - |
| Tetrahydrocortisone (p, b) | **364.2222** | **9.3** | -8 | **ESI(+)** | b | monoisotopic mass + isotopic pattern distrubution | - |
| Dihydrocortisol (p, b) | **364.2228** | **9.2** | -6 | **ESI(+)** | b | monoisotopic mass + isotopic pattern distrubution | - |
| Ferrulic acid 4-O-glucuronide (n, a) | **370.0883** | **7.4** | -5 | **ESI(-)** | a | 59.015, 175.0242, 193.0501 (+standard) | HMDB |
| Dihydroferulic acid 4-O-glucuronide (n,a) | **372.1039** | **7.5** | -5 | **ESI(-)** | a | 59.0139,175.0248, 177.0555, 195.0663, 309.0980 | HMDB |
| Tetrahydrofolic acid (n, b) | **445.1806** | **11.2** | 22 | **ESI(-)** | b | monoisotopic mass + isotopic pattern distrubution | - |
| Stachyose (n, a) | **666.2201** | **1.1** | -3 | **ESI(-)** | a | 179.0551, 221.0660, 341.1181, 383.1194, 485.1516 | Metlin |
| beta-D-Mannosylphosphodecaprenol (n, b) | **940.6469** | **9.6** | -9 | **ESI(-)** | b | monoisotopic mass + isotopic pattern distrubution | - |
