# Supplementary material for: LC-QTOF-MS and 1H NMR Metabolomics Verifies Potential Use of Greater Omentum for Klebsiella pneumoniae Biofilm Eradication in Rats
Source: Pathogens. 2020 May 21;9(5):399. doi: 10.3390/pathogens9050399 (PMC7281169; doi:10.3390/pathogens9050399)
Supplement: Supplementary file 1 [file pathogens-09-00399-s001.zip › Supplementary data PROOF/Table S2_PROOF.docx]

**Table S2**. List of annotated metabolites significantly different between the studied groups: sterile omentum versus controls (SO vs. C), sterile peritoneum versus controls (SP vs. C), infected omentum versus controls (IO vs. C), infected peritoneum versus controls (IP vs. C).

| **day** | d0 |  | d0 |  | d2 |  | d2 |  | d5 |  | d5 |  | d0 |  | d0 |  | d2 |  | d2 |  | d5 |  | d5 |  |
| --- | --- | --- | --- | --- | --- | --- | --- | --- | --- | --- | --- | --- | --- | --- | --- | --- | --- | --- | --- | --- | --- | --- | --- | --- |
| **groups** | **SO_C** |  | **SP_C** |  | **SO_C** |  | **SP_C** |  | **SO_C** |  | **SP_C** |  | **IO_C** |  | **IP_C** |  | **IO_C** |  | **IP_C** |  | **IO_C** |  | **IP_C** |  |
| **TCA CYCLE, CoA Biosynthesis** |  |  |  |  |  |  |  |  |  |  |  |  |  |  |  |  |  |  |  |  |  |  |  |  |
| N-acetylaspartate *(p, a)* | -12% | - | 6% | - | 50% | * | 113% | * | 40% | - | -8% | - | -7% | - | 19% | - | 100% | ** | 128% | * | -7% | - | 8% | - |
| L-Lactic acid *(m)* | -37% | - | -95% | ** | -32% | - | -95% | ** | 2% | - | -67% | - | -92% | ** | -91% | ** | -73% | * | -93% | ** | -24% | - | -34% | - |
| Formic acid *(m)* | -36% | - | 42% | - | 17% | - | -14% | - | -6% | - | -78% | * | -41% | - | -48% | - | -36% | * | -33% | * | -21% | - | -41% | - |
| Acetic acid *(m)* | -32% | - | -90% | ** | -29% | - | -86% | ** | 0% | - | -85% | * | -87% | ** | -88% | ** | -61% | * | -87% | ** | -23% | - | -45% | - |
| Pyruvic acid *(m)* | -25% | * | -53% | ** | -31% | * | -58% | ** | -31% | - | -60% | * | -42% | * | -50% | ** | -46% | ** | -58% | ** | -9% | - | -43% | - |
| Citric acid *(m)* | 7% | - | -21% | - | -23% | * | -41% | ** | 15% | - | 398% | * | -4% | - | -20% | - | -30% | - | -40% | ** | 231% | - | 218% | - |
| alpha-ketoglutaric acid *(m)* | 14% | - | -35% | * | -32% | * | -57% | ** | -15% | - | -28% | - | 1% | - | -30% | * | -23% | - | -50% | * | 29% | - | -30% | - |
| cis-Aconitic acid *(m)* | -1% | - | 22% | - | -12% | - | -10% | - | -10% | - | -20% | - | 18% | - | -6% | - | 0% | - | -6% | - | -3% | - | -25% | * |
| trans-Aconitic acid *(m)* | -11% | - | 5% | - | -8% | - | 2% | - | -25% | - | -23% | - | 15% | - | 14% | - | 2% | - | -1% | - | -23% | - | -18% | - |
| Succinic acid *(m)* | -11% | - | -46% | ** | -34% | - | -59% | * | 23% | - | -66% | * | -37% | * | -59% | ** | -44% | * | -62% | ** | 1% | - | -57% | * |
| Succinic acid semialdehyde *(n, a)* | 10% | - | -57% | * | -41% | - | -74% | * | -46% | - | -24% | - | -11% | - | -27% | - | -47% | * | -63% | ** | 50% | - | -17% | - |
| Fumaric acid *(m)* | 8% | - | 77% | - | -48% | ** | -34% | ** | -27% | - | 134% | * | 115% | - | 53% | - | -2% | - | -38% | * | 31% | - | 70% | - |
| Malic acid *(n, a)* | -2% | - | 25% | - | -31% | - | -29% | * | 21% | - | 38% | - | 40% | - | 22% | - | 13% | - | -28% | - | 51% | - | -1% | - |
|  |  |  |  |  |  |  |  |  |  |  |  |  |  |  |  |  |  |  |  |  |  |  |  |  |
| **AMINO ACIDS** |  |  |  |  |  |  |  |  |  |  |  |  |  |  |  |  |  |  |  |  |  |  |  |  |
| Pyroglutamic acid *(n, b)* | -22% | - | -9% | - | 41% | - | 88% | - | 14% | - | 4% | - | -31% | - | -6% | - | 94% | * | 70% | * | 37% | - | -9% | - |
| L-Alanine *(m)* | -20% | - | -50% | ** | -20% | - | -68% | ** | 49% | - | -76% | * | -51% | ** | -54% | ** | -52% | * | -66% | ** | -42% | - | -42% | - |
|  |  |  |  |  |  |  |  |  |  |  |  |  |  |  |  |  |  |  |  |  |  |  |  |  |
| **TRYPTOPHAN METABOLISM** |  |  |  |  |  |  |  |  |  |  |  |  |  |  |  |  |  |  |  |  |  |  |  |  |
| Tryptophyl-Glutamate *(n, b)* | 0% | - | -20% | - | -29% | - | -63% | * | 13% | - | -35% | - | 14% | - | -22% | - | -38% | * | -52% | * | -12% | - | -38% | - |
| Indoxyl sulfate *(n, a)* | 113% | * | 110% | * | 35% | - | -21% | - | -12% | - | -34% | - | 74% | - | 47% | - | 12% | - | 33% | - | -8% | - | -33% | - |
|  |  |  |  |  |  |  |  |  |  |  |  |  |  |  |  |  |  |  |  |  |  |  |  |  |
| **HIPPURATE BIOSYNTHESIS** |  |  |  |  |  |  |  |  |  |  |  |  |  |  |  |  |  |  |  |  |  |  |  |  |
| Benzoic acid *(m)* | -8% | - | -94% | ** | -31% | - | -93% | ** | 12% | - | -59% | - | -88% | ** | -92% | ** | -72% | ** | -92% | ** | -31% | - | -31% | - |
| Glycine *(m)* | -9% | - | -88% | ** | -24% | - | -86% | ** | 6% | - | -50% | - | -81% | ** | -83% | ** | -66% | * | -87% | ** | -32% | - | -31% | - |
| Hippuric acid *(m)* | 7% | - | ##### | ** | 111% | - | 994% | ** | -9% | - | 868% | * | 948% | ** | ##### | ** | 656% | - | 855% | ** | 372% | - | 327% | - |
|  |  |  |  |  |  |  |  |  |  |  |  |  |  |  |  |  |  |  |  |  |  |  |  |  |
| **CHOLINE, LIPID METABOLISM** |  |  |  |  |  |  |  |  |  |  |  |  |  |  |  |  |  |  |  |  |  |  |  |  |
| Choline *(p, a)* | 33% | - | -46% | - | 24% | - | -48% | * | 277% | * | -13% | - | 22% | - | -16% | - | -59% | * | -46% | * | 250% | - | 186% | - |
| Betaine *(m)* | 0% | - | -17% | - | -42% | - | -53% | * | -42% | - | -45% | - | -22% | - | -32% | * | -59% | ** | -57% | ** | -23% | - | -47% | - |
| Creatinine *(p, a)* | -12% | - | 30% | - | 42% | - | 58% | * | -17% | - | 65% | * | 30% | - | 34% | - | 49% | - | 27% | - | 50% | - | 79% | * |
| Phosphorylcholine *(p,b)* | 89% | * | 315% | * | 14% | - | 87% | - | -74% | - | 36% | - | 187% | - | 182% | - | 55% | - | 36% | - | -12% | - | -31% | - |
| Tetrahydrofolic acid *(n, b)* | -37% | * | -60% | * | -13% | - | 1% | - | -8% | - | -6% | - | -37% | * | -4% | - | -13% | - | -4% | - | 52% | - | 3% | - |
|  |  |  |  |  |  |  |  |  |  |  |  |  |  |  |  |  |  |  |  |  |  |  |  |  |
| **ACYL GLYCINES** |  |  |  |  |  |  |  |  |  |  |  |  |  |  |  |  |  |  |  |  |  |  |  |  |
| Isonicotinylglycine *(p, a)* | 47% | * | 324% | * | 95% | - | 276% | - | na | - | na | - | 232% | * | 150% | - | 240% | - | 215% | - | na | - | na | - |
| Phenylacetylglycine *(p, a)* | 29% | - | 163% | * | 44% | - | 84% | - | -80% | - | 287% | - | 99% | - | 71% | - | 99% | - | 157% | - | 125% | - | 127% | - |
| 3-Hydroxyhippuric acid *(n, a)* | 229% | - | 436% | - | 106% | - | 359% | * | 110% | - | 722% | - | 484% | * | 498% | * | 372% | - | 353% | - | ##### | - | ##### | - |
|  |  |  |  |  |  |  |  |  |  |  |  |  |  |  |  |  |  |  |  |  |  |  |  |  |
| **HORMONES** |  |  |  |  |  |  |  |  |  |  |  |  |  |  |  |  |  |  |  |  |  |  |  |  |
| Tetrahydrocortisone *(p, b)* | -2% | - | 3% | - | 71% | - | 61% | - | -12% | - | -10% | - | 13% | - | 37% | - | 101% | * | 131% | * | -19% | - | -16% | - |
| Dihydrocortisol *(p, b)* | -5% | - | 30% | - | 47% | - | 100% | * | 37% | - | 42% | - | 49% | - | 37% | - | 97% | - | 140% | * | -27% | - | -23% | - |
|  |  |  |  |  |  |  |  |  |  |  |  |  |  |  |  |  |  |  |  |  |  |  |  |  |
| **DIETARY METABOLITES** |  |  |  |  |  |  |  |  |  |  |  |  |  |  |  |  |  |  |  |  |  |  |  |  |
| Taurine *(p, a)* | -56% | * | -30% | - | -50% | * | -59% | * | -60% | * | 5% | - | -51% | ** | -51% | ** | -61% | ** | -49% | * | -28% | - | -8% | - |
| Indolylacryloylglycine *(n, a)* | -25% | - | -40% | - | -49% | - | -53% | - | -15% | - | 42% | - | -29% | - | -51% | - | -45% | * | -61% | * | 74% | - | 28% | - |
| kamlolenic acid *(p, b)* | -54% | - | -30% | - | -14% | - | -45% | * | 13% | - | -43% | - | 12% | - | -6% | - | -20% | - | -26% | - | 41% | - | 47% | - |
| Xylitol (+Ribitol) *(n, a)* | -9% | - | -7% | - | 30% | - | 17% | - | -58% | - | -37% | * | 6% | - | 7% | - | 29% | - | 58% | - | -27% | - | -34% | * |
| Stachyose *(n, a)* | -32% | - | -75% | * | -3% | - | -32% | - | 107% | - | 20% | - | -23% | - | 85% | - | -42% | - | -21% | - | -20% | - | 19% | - |
|  |  |  |  |  |  |  |  |  |  |  |  |  |  |  |  |  |  |  |  |  |  |  |  |  |
| **POLYPHENOLS INTAKE** |  |  |  |  |  |  |  |  |  |  |  |  |  |  |  |  |  |  |  |  |  |  |  |  |
| Phenol sulphate *(n, a)* | 28% | - | 93% | * | 33% | - | 15% | - | -70% | - | -12% | - | 40% | - | 5% | - | 22% | - | 22% | - | -53% | - | -57% | - |
| Tyrosol 4-sulfate *(n, b)* | 127% | * | 218% | * | -15% | - | -5% | - | 0% | - | -15% | - | 138% | ** | 186% | ** | -28% | - | -36% | - | -12% | - | -49% | - |
| Pyrocatechol sulphate *(n, a)* | 20% | - | 74% | * | -12% | - | 32% | * | 0% | - | 12% | - | 13% | - | 11% | - | 21% | - | 21% | - | -14% | - | -3% | - |
|  |  |  |  |  |  |  |  |  |  |  |  |  |  |  |  |  |  |  |  |  |  |  |  |  |
| **URIC ACID** |  |  |  |  |  |  |  |  |  |  |  |  |  |  |  |  |  |  |  |  |  |  |  |  |
| Allantoin *(n, a)* | -17% | - | -30% | - | 14% | - | 6% | - | 27% | - | 118% | - | 8% | - | 2% | - | 20% | - | 19% | - | 100% | - | 75% | - |
|  |  |  |  |  |  |  |  |  |  |  |  |  |  |  |  |  |  |  |  |  |  |  |  |  |
| **CAFFEIC ACID FERRULIC ACID** |  |  |  |  |  |  |  |  |  |  |  |  |  |  |  |  |  |  |  |  |  |  |  |  |
| Caffeic acid 3-sulfate / Caffeic acid 4-sulfate *(p, a)* | 41% | * | 47% | * | -5% | - | -14% | - | 48% | - | -12% | - | 23% | - | 12% | - | -5% | - | -27% | - | 26% | - | 20% | - |
| Dihydrocaffeic acid 3-sulfate *(n, a)* | 63% | - | 242% | * | -1% | - | 89% | * | 12% | - | 57% | - | 155% | * | 121% | - | 49% | - | 44% | - | 8% | - | -12% | - |
| Ferulic acid *(n, a)* | 12% | - | 220% | * | 9% | - | 36% | * | -11% | - | -4% | - | 92% | * | 92% | - | 30% | - | 30% | - | -14% | - | -37% | * |
| Ferulic acid 4-O-sulfate *(n, b)* | 33% | - | 268% | * | 32% | - | 78% | * | -14% | - | 3% | - | 85% | * | 105% | - | 72% | ** | 34% | - | -14% | - | -33% | * |
| Dihydroferulic acid *(n, a)* | -49% | - | -86% | * | -66% | - | -91% | * | 0% | - | -9% | - | -77% | ** | -79% | ** | -46% | - | -91% | ** | 57% | - | -33% | - |
| Ferrulic acid 4-O-glucuronide *(n, a)* | 133% | - | 565% | - | 326% | - | 387% | - | -38% | - | 355% | - | 541% | * | 448% | * | 459% | * | 437% | ** | 60% | - | 14% | - |
| Dihydrocaffeic acid 3-O-glucuronide *(n, b)* | 116% | - | 354% | * | 168% | - | 565% | * | -97% | - | 308% | - | 305% | * | 233% | * | 260% | - | 394% | * | 184% | - | 183% | - |
| Dihydroferulic acid 4-O-glucuronide *(n, b)* | 125% | - | 398% | * | 239% | - | 611% | * | -41% | - | 290% | - | 341% | ** | 265% | ** | 441% | * | 532% | ** | 340% | - | 131% | - |
| Dihydroferuloylglycine *(p, b)* | -11% | - | 258% | * | 252% | - | 146% | - | -34% | - | -2% | - | 194% | * | 87% | - | 233% | - | 158% | * | 62% | - | -30% | - |
|  |  |  |  |  |  |  |  |  |  |  |  |  |  |  |  |  |  |  |  |  |  |  |  |  |
| **OTHERS** |  |  |  |  |  |  |  |  |  |  |  |  |  |  |  |  |  |  |  |  |  |  |  |  |
| Urea *(m)* | 1% | - | 49% | ** | -9% | - | 4% | - | -28% | - | -37% | - | 24% | - | 12% | - | 4% | - | 12% | - | -31% | - | -55% | * |
| Histamine *(p, a)* | 29% | - | 114% | - | 28% | - | 20% | - | -23% | - | 69% | - | 75% | ** | 50% | * | 63% | ** | 49% | * | 17% | - | 4% | - |
| 17,21-Dihydroxypregnenolone *(p, b)* | 8% | - | 130% | - | 90% | - | 118% | - | 1% | - | 18% | - | 93% | - | 109% | - | 263% | * | 381% | * | -2% | - | 31% | - |
| Isohomovanillic acid *(n, a)* | 20% | - | 36% | * | -4% | - | -41% | - | 12% | - | -12% | - | 34% | - | 16% | - | 25% | - | -30% | - | -8% | - | -30% | - |
| 4-Hydroxybenzaldehyde *(n, a)* | 20% | - | -26% | - | -30% | - | -50% | - | 86% | - | 99% | - | -16% | - | -41% | ** | -30% | - | -39% | - | 48% | - | 67% | - |
| 3-Hydroxydodecanedioic acid *(n, b)* | 0% | - | -34% | * | -13% | - | -50% | * | 0% | - | -46% | * | -6% | - | -36% | * | -37% | ** | -49% | ** | -35% | - | -47% | * |
| 3-Hydroxyisoheptanoic acid /Ethyl 2-hydroxyisovalerate *(n, b)* | 31% | - | 3% | - | -13% | - | -60% | * | -16% | - | -56% | - | 51% | - | 71% | - | -25% | - | -39% | * | -28% | - | -29% | - |
| O-methoxycatechol-O-sulphate *(n, b)* | 28% | - | 125% | * | 45% | - | 63% | - | 8% | - | 85% | - | 41% | - | 19% | - | 55% | - | 79% | * | -11% | - | -6% | - |
| Trigonelline *(m)* | -10% | - | 36% | ** | -19% | - | 15% | - | -4% | - | -4% | - | 14% | - | 14% | - | -3% | - | -10% | - | -5% | - | -14% | - |
| Oxolan-3-one *(n, a)* | -4% | - | 29% | * | 6% | - | 8% | - | 19% | - | 18% | - | 29% | ** | 15% | - | 26% | ** | 17% | * | 22% | - | 10% | - |
| N-Acetyl-7-O-acetylneuraminic acid *(n, a)* | -7% | - | -15% | - | -8% | - | -40% | * | -6% | - | -48% | - | 86% | - | -22% | - | -53% | * | -41% | * | 1% | - | -24% | - |
| Ethanol *(m)* | -6% | - | -8% | - | -19% | - | -20% | - | -21% | - | -44% | * | -2% | - | 9% | - | -16% | - | -17% | - | 26% | - | -9% | - |
| 2-Octenedioic acid / cis-4-Octenedioic acid / trans-3-Octenedioic acid *(n, b)* | -14% | - | -62% | * | 17% | - | 36% | - | -5% | - | -7% | - | -20% | - | -4% | - | 64% | - | 61% | - | 65% | - | 15% | - |
| beta-D-Mannosylphosphodecaprenol *(n, b)* | 72% | - | -61% | * | 23% | - | -59% | * | -1% | - | -48% | - | -45% | * | -7% | - | -32% | - | -44% | * | -6% | - | 6% | - |

Percentage change between the two groups was calculated using following equation: 100% * (group 1 - group 2)/group 2. *p* values: **p*value < 0,05; ** *p* value < 0,01; - *p* value not significant. *(n)*  metabolites found in negative polarity mode; *(p)*metabolites found in positive polarity mode; *(m)* metabolites found in **^1^**H NMR. *(a)* metabolites annotated by MS/MS spectra and MS fragmentation pattern; *(b)*metabolites annotated putatively by exact mass data and isotopic pattern distribution. Color coding: red- increase in the first comparing to the second group, light blue-decrease in the first comparing to the second , yellow – denotes statistically significant change.
